# The complete mitochondrial genome of *Agelena silvatica* (Araneae: Agelenidae)

**DOI:** 10.1080/23802359.2017.1280702

**Published:** 2017-02-02

**Authors:** Xiao-Lin Zhu, Zhi-Sheng Zhang

**Affiliations:** a Key Laboratory of Eco-environments in Three Gorges Reservoir Region (Ministry of Education), School of Life Sciences, Southwest University, Chongqing, P. R. China;; b Key Laboratory of Freshwater Fish Reproduction and Development (Ministry of Education), School of Life Sciences, Chongqing, P. R. China

**Keywords:** *Agelena silvatica*, mitochondrial genome, phylogenetic relationship

## Abstract

The complete mitochondrial genome sequence of a funnel-web weaving spider, *Agelena silvatica* (Agelenidae), was obtained using long PCR and conventional PCR methods. The circular genome is 14,776 bp in length. It contains 13 protein-coding genes, 22 transfer RNAs, 2 ribosomal RNAs, and a control region. The A + T content (74.46%) of the all base composition is higher than G + C content (25.54%). Bayesian and maximum likelihood phylogenetic analyses demonstrate that *Agelena silvatica* is the sister clade of the water spider, *Argyroneta aquatica*.

The funnel-web weaving spider belongs to the family Agelenidae, a large family in Araneae, owning about 1200 species all over the world (World Spider Catalog 2016). The species, *Agelena silvatica* Oliger, 1983 (Araneae: Agelenidae), previously known as *A. limbata* Thorell, 1897 since Bösenberg and Strand (1906), is a common species in East Asia (found in South China, Korea, Japan) and Far East of Russia (Zhang et al. 2005). Generally, this species has large body size (∼10–20 mm), constructs a typical funnel web on shrubs or trees to trap insect or other animals (Tanaka 1989). Studies on ecology, physiology, and behaviour of this species have been carried out well (Tanaka 1984, 1991, 1995; Masumoto 1994; Park & Moon 2002). But, to date, only a few molecular data of this species are characterized. Fang et al. (2000) analyzed the phylogeny of spiders based on two partial mitochondrial genes (16S and 12S rRNAs). Here, the complete mitochondrial genome sequence of *A. silvatica* was determined and used for the phylogenetic analysis of spiders. This is the first report for the complete mitochondrial genome sequence of agelenid spiders.

Specimens of *A. silvatica* were collected from the Jinyun Mountain Nature Reserve in Beibei (29°50.192′ N, 106°23.751′ E, Alt. 749m), Chongqing of China in July 2015, and preserved in the Arachnological laboratory of Prof. Zhisheng Zhang (School of Life Sciences, Southwest University). Three short DNA fragments were amplified and sequenced using universal primers (Simon et al. 1994; Vrijenhoek 1994). Subsequently, based on the acquired sequences, five pairs of species-specific primers were designed to amplify long fragments, and the long-PCR products were sequenced by primer-walking methods. After assembling and alignment, the complete mitogenome sequence of *A. silvatica* was submitted to GenBank with an accession number KX290739.

The complete mitogenome of *A. silvatica* is 14,776 bp in length, and composed of 22 tRNA genes, 13 protein-coding genes, 2 rRNA genes, and a control region. Total basic composition of the mitochondrial genome is 31.17% A, 43.29% T, 8.91% C and 16.63% G. The A + T content (74.46%) was greatly higher than the G + C content (25.54%).

In all 13 protein-coding genes, five kinds of initiation codons were used (ATA, TTA, TTG, ATT, and ATG). Among those genes, six protein-coding genes begin with ATA (nad1, nad2, nad3, nad5, nad4 and nad4L). The cox1 gene begins with TTA. The cox2 and cox3 start with TTG. Start codon ATT is found in atp8 and cob. ATG begins for atp6 and nad6. Six protein-coding genes are terminated with TAA (nad2, cox1, cox2, atp6, nad3, and nad6). Three are ended with TAG (cox3, atp8 and nad1). Incomplete stop codons (T––) are found in nad4, nad4L, nad5, and cob. The 12S and 16S ribosomal RNA genes are 697 and 1028 bp, respectively. The length of the 22 tRNA genes ranged from 39 to 85 bp.

In addition, 13 protein-coding genes were used to study the phylogenetic relationships of spiders by performing Bayesian inference (BI) and maximum likelihood (ML) algorithms. The sequences of representative species were downloaded from GenBank. *Heptathela hangzhouensis* and *Liphistius erawan* were set as outgroups.

BI analysis was performed using MrBayes v.3.1 (Ronquist & Huelsenbeck 2003), and ML analysis was performed using RAxML v.7.0.4 (Stamatakis 2006). In BI and ML trees, the ingroups are divided into two clades, Haplogynae and Entelegynae, which are consistent with the cladogram in Coddington (2005). *A. silvatica* forms a sister relationship with *Argyroneta aquatica* (0.98 in BI and 52 in ML) (Figure 1).

**Figure 1.**
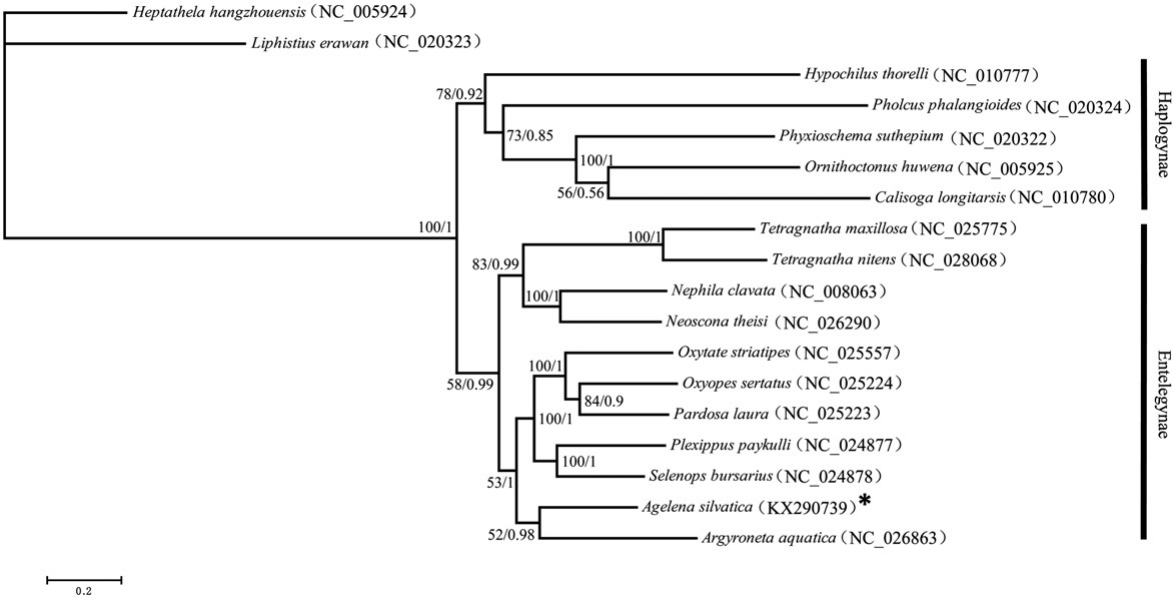
The cladogram of Araneae.
